# The Therapeutic Potential of Exosomes in Soft Tissue Repair and Regeneration

**DOI:** 10.3390/ijms23073869

**Published:** 2022-03-31

**Authors:** Rou Wan, Arif Hussain, Atta Behfar, Steven L. Moran, Chunfeng Zhao

**Affiliations:** 1 Division of Plastic Surgery, Mayo Clinic, Rochester, MN 55905, USA; wan.rou@mayo.edu (R.W.); hussain.arif@mayo.edu (A.H.); moran.steven@mayo.edu (S.L.M.); 2 Department of Cardiovascular Medicine, Mayo Clinic, Rochester, MN 55905, USA; behfar.atta@mayo.edu; 3 Van Cleve Cardiac Regenerative Medicine Program, Center for Regenerative Medicine, Mayo Clinic, Rochester, MN 55905, USA; 4 Department of Molecular Pharmacology and Experimental Therapeutics, Mayo Clinic, Rochester, MN 55905, USA; 5 Department of Orthopedic Surgery, Mayo Clinic, Rochester, MN 55905, USA

**Keywords:** exosomes, soft tissue healing, skeletal muscle healing, tendon healing, peripheral nerve healing, repair and regeneration, extracellular vesicles, therapeutic applications

## Abstract

Soft tissue defects are common following trauma and tumor extirpation. These injuries can result in poor functional recovery and lead to a diminished quality of life. The healing of skin and muscle is a complex process that, at present, leads to incomplete recovery and scarring. Regenerative medicine may offer the opportunity to improve the healing process and functional outcomes. Barriers to regenerative strategies have included cost, regulatory hurdles, and the need for cell-based therapies. In recent years, exosomes, or extracellular vesicles, have gained tremendous attention in the field of soft tissue repair and regeneration. These nanosized extracellular particles (30–140 nm) can break the cellular boundaries, as well as facilitate intracellular signal delivery in various regenerative physiologic and pathologic processes. Existing studies have established the potential of exosomes in regenerating tendons, skeletal muscles, and peripheral nerves through different mechanisms, including promoting myogenesis, increasing tenocyte differentiation and enhancing neurite outgrowth, and the proliferation of Schwann cells. These exosomes can be stored for immediate use in the operating room, and can be produced cost efficiently. In this article, we critically review the current advances of exosomes in soft tissue (tendons, skeletal muscles, and peripheral nerves) healing. Additionally, new directions for clinical applications in the future will be discussed.

## 1. Introduction

Soft tissue injuries, including skin, muscle, tendon, and nerve injury, affect millions of people every year [1,2,3,4,5,6]. Studies on tissue repair and regeneration have focused primarily on skin wound healing; however, for complete repair of composite soft tissue defects, healing of tendons, skeletal muscles, and peripheral nerves will also be required to restore function. Thus, the healing of composite defects requires more attention from researchers.

One of the current novel strategies in regenerative medicine is cell-based therapy. Although cell-based therapy has clear beneficial effects on tissue repair and regeneration, it still has a number of drawbacks, such as low survival rate of the cells, the decreased regenerative capacity of engrafted cells, immune-mediated rejection, risk of capillary blockade during infusion, tumorigenesis, and ethical and regulatory concerns, inhibiting its wide application in clinical settings [7,8,9,10,11]. An alternative to cell-based therapy is the use of exosomes. These extracellular vesicles can not only produce the regenerative benefits of stem cells but also have lower immunogenicity, simpler storage and transportation, and more affordable cost compared with live stem cell therapies [12,13,14,15,16]. Thus, interests in exosomes, a potential cell-free biotherapeutic, have expanded rapidly in the medical society in recent years due to their diverse pathological and therapeutic effects, suggesting great therapeutic potential [6,17].

Exosomes are a subtype of extracellular vehicle (EV) measuring 30 nm to 200 nm in diameter [18], and are formed from the inward budding of the endosome membrane [12,19,20,21]. First discovered in 1983 by Stahl et al., these particles were later termed “exosomes” [22]. They are of endocytic origin, and released extracellularly with functional properties from their original cells, enclosed in a single outer membrane [18,23]. When intracellular multivesicular bodies (MVBs), initially early endosomes, form intraluminal vesicles (ILVs) and fuse with the cell membrane, they secrete substances within the cells extracellularly in the form of vesicles, which are known as exosomes [9,12,19,24]. Therefore, the structure and biological composition of exosomes, including proteins, peptides, lipids, nucleic acids, and other metabolites, are highly related to their origins [18,19,23]. Exosomes in history were only considered to remove unwanted cellular waste from the cells [18,19]. More recent studies have demonstrated that they indeed play a critical role in the pathogenesis and progresses of many diseases (e.g., cancer, cardiovascular diseases, musculoskeletal disease, infection, and neurodegenerative diseases), and can potentially facilitate treatment delivery to desired targets [9,12,19,21,25,26]. The delivery system run by exosomes is also highly stable due to the lipid bilayer that protects their cargo from enzymatic degradation.

Almost all cell types secrete exosomes, and they are widespread in plasma, saliva, urine, cerebrospinal fluid, ascites, and other biofluids [9]. Tissue regeneration largely relies on a series of highly coordinated events orchestrated between multiple cell types. Exosomes are well known for playing the leading role in intercellular communication during this process. They can be incorporated into targeted cells after being released through mechanisms including endocytosis, membrane fusion, and receptor-mediated interaction, thereby transferring their contents (proteins, mRNAs, miRs, lipids, metabolites, and other cytosol components) [27,28,29]. The literature has shown that the endosomal sorting complexes required for transport (ESCRT) pathway and Rab GTPases are the key mediators [9,19,30,31,32,33]. Thus, ESCRT proteins (Alix, TSG101, HSC70, and HSP90b) can be seen as exosomal markers [8,19]. However, some studies have also recently established an ESCRT-independent mechanism [19,30,34,35,36,37,38,39,40,41]. The exact mechanisms of MVB regulation and exosome release and targeting are still not fully understood.

The use of exosomes as a therapeutic approach is greatly limited by the present isolation and purification techniques for producing large-scale and high-quality exosomes. There are seven currently performed exosome isolation techniques: differential centrifugation, precipitation, flushing separation, ultrafiltration, antibody affinity capture, microfluidic separation, and mass spectrometry [42]. With differential centrifugation considered as the “gold standard”, the disadvantage of consuming a large amount of time and samples is still present [42]. None of the present methods can isolate exosomes from other EVs quickly, reliably, or on a large scale [42,43]. Indeed, the low productivity of exosomes is one of the main obstacles for translating exosome-based therapeutics to clinical settings. In practice, the sizes and markers overlap among different types of EVs [21,44], and identifying an exosome’s endosomal origin can be challenging after it leaves the parent cells. Along with the immature technology of exosome purification [9,45], the term “extracellular vesicles/particles” is sometimes appropriate, and is used when the strict definition of exosomes cannot be confirmed or when exosomes are mixed with other types of EVs [9,12,46,47,48]. In this review, both EVs and exosomes indicate exosomes. In order to better define exosome characteristics and assist downstream application, various techniques for characterization of exosomes are applied, including electron microscopy, atomic force microscopy, nanoparticle tracking analysis, dynamic light scattering, tunable resistive pulse sensing, Western blotting and ELISA, qRT-PCR, and flow cytometry. Other issues that need further exploration include long-term storage, in vivo stability, and strategies for tissue-specific targeting and delivery of exosomes [9,42,49,50,51]. This paper reviews the effects of exosomes on tendon, skeletal muscle, and peripheral nerve repair and regeneration, and highlights the related bio-engineered applications.

## 2. Exosomes in Tendon and Repair and Regeneration

### 2.1. Regulating Tendon Extrinsic Healing

There are two main proposed mechanisms of tendon healing: intrinsic and extrinsic. During extrinsic healing, inflammatory cells and fibroblasts migrate from surrounding tissues, such as the sheath and synovium, to react in the initial inflammatory phase in the healing process [52,53]. Growth factors and cytokines released by the inflammatory cells during the initial phase play a dominant role in directing stem cell differentiation, tenocyte proliferation, and neo-tendon maturation in the later proliferative and regenerative phase [54,55,56,57]. Existing studies have shown encouraging anti-inflammatory and anti-scarring properties of exosomes in extrinsic healing of tendons. One of the most well-recognized mechanisms of exosomes is their capability of facilitating macrophage polarization and maintaining a dominant M2 pattern via paracrine [57,58,59,60,61,62,63,64,65,66].

M2 phenotype macrophages are associated with anti-inflammation and the rebuilding of the extracellular matrix. A lower M1/M2 macrophage ratio indicates subsequent benefits in tendon tissue remodeling [58,64,66]. In a seminal study, Sprague Dawley rats with patellar tendon injuries were treated with fibrin-containing exosomes derived from rat bone marrow stromal cells (BMSC-Exos). The researchers found significantly increased mRNA expression of M2 macrophage stimulators (IL-4 and IL-10), along with decreased expression of M1 macrophages markers (IFNγ, IL-1B, and IL-6) in the BMSC-EV group, both in vitro and in vivo. The number of CCR7-M1 macrophages in the newly formed tendon tissue at the repair site of the rats was significantly greater in the groups without exosomal treatment [59]. Zhang et al. confirmed the same observation in vivo on Sprague Dawley rats with Achilles tendon injuries after treatment of exosomes derived from rat tendon stem cells (TSC-Exos) [63]. Hence, exosomes improved tendon healing by upregulating anti-inflammatory cytokines and polarizing macrophages toward the CD163+ M2 phenotype. Consistently, Liu et al. extracted rat ADSC-Exos, and found significantly increased CD163+ M2 macrophages after ADSC-Exo treatment in vivo [57].

Additionally, several studies have indicated that exosomes inhibit the expression of various pro-inflammatory mediators and cytokines, including NF-kB, IL-1β, IL-6, IL-8, IL-18, COX-2, MMP1, MMP3 MMP9, iNOS, CXCL, IFNγ, and TNFα, leading to attenuated inflammation, which is ideal for tendon regeneration [56,57,59,61,63,67,68,69,70,71,72,73,74]. Zhang et al. observed dramatically suppressed COX-2 factor in the TSC-Exos treatment group [63]. Shen et al. recorded reduced expression and activity of NF-kB, proinflammatory cytokine IL-1b, and the major collagenase MMP1 at the injury site in a mouse Achilles tendon healing model after treatment with ASC-Exos, facilitating anabolic tissue response for tendon matrix regeneration [61]. Zhang et al. harvested tenocytes from the supraspinatus tendons of patients with chronic rotator cuff tears. The team suggested that the decreased synthesis of proinflammatory cytokines might have been achieved by the AMPK signaling pathway, suppressing Wnt/b-catenin activity [73]. Studies have revealed that diseased tendons possessed stromal cells with elevated expressions of proinflammatory genes compared with healthy tendons [56,75,76]. Therefore, triggering and augmenting the expression of various anti-inflammatory or pro-resolving mediators is fundamentally beneficial for tendon repair and regeneration.

### 2.2. Enhancing Tendon Intrinsic Healing

Intrinsic healing of the tendon mainly involves the differentiation of tendon stem cells (TSCs) and the proliferation and migration of tenocytes around the injured site, the processes of which take place in the proliferative or reparative phase of tendon healing. This intrinsic healing process is extremely important for maintaining and restoring biomechanical functions after an injury [52,77].

Cui et al. treated tenocytes from murine superficial flexor tendons with mouse bone marrow-derived macrophages exosomes (BMDM-Exos), and demonstrated increased tenocyte proliferation and migration in vitro [78]. Shi et al. used CD146+ to mark TSCs, and found an induced accumulation of CD146+ cells at the injury site after BMSC-EV treatment, whereas no apparent CD146 staining was present in other groups [59]. Moreover, cleaved caspase-3 signals were decreased in the BMSC-EV group, indicating reduced apoptotic cell death in the tendon [59]. One popular hypothesis of the mechanism of exosome-induced tenogenesis is TGF-b dependent signaling [79]. Xu et al. found that tenocyte-derived exosomes (tenosomes) can induce the tenogenic differentiation of mesenchymal stem cells in a dose-dependent manner, and reported that tenosomes contain a higher level of TGF-b than tenocytes. To further prove their finding, the team applied a TGF-b signaling inhibitor (SB 431542) during the exosomal treatment, and found an abolished tenogenic effect on MSCs from tenosomes. They also suggested that TGF-b signaling played a critical role in initiating the expression of scleraxis (SCX), an important tenocyte marker, in MSCs [79]. Consistently, Li et al. demonstrated that BMSC-Exos greatly increased proliferation and migration of tenocytes, and significantly upregulated TGF-b1 in BMSC-Exos, as compared to normal BM-MSCs. Inhibiting TGF-b1 signaling reversed all of the effects of enhanced cell proliferation, cell migration, and levels of tenogenic genes from BMSC-Exo treatment [80]. Another study similarly observed that the TSC-Exo possessed a great amount of TGF-b, and exerted tendon healing properties via a TGF-b dependent pathway. Additionally, they revealed that TGF-b from exosomes activated the TGF b-Smad2/3 signaling pathway and the extracellular signal-regulated kinase (ERK)1/2 signaling pathway of TSCs [81]. The former pathway can increase the expression of MMP2, and the latter pathway is related to tenocyte proliferation [81]. Liu et al. also published the activation of SMAD2/3 signaling by ADSC-Exos in the promotion of tendon healing [57]. Interestingly, in a canine ex vivo model, using a purified exosome TISSEEL patch showed significantly higher amounts of tenocytes and improved biomechanical properties of the tendon, but a reduction in the expression of TGF-b [74].

Other pathways that have been published include the mTOR signaling, SMAD1/5/9, PI3K/AKT, MAPK/ERK1/2, phosphorylated AMPKa, and Wnt/b-catenin. Yao et al. used a transforming growth factor-β1 (TGF-β1) inhibitor (SB-431542) and an mTOR inhibitor (rapamycin) with HUMSC-Exo treatment in a rat Achilles tendon injury model, and displayed that the PTEN/mTOR/TGF-β1 signaling cascades played beneficial roles in HUMSC-Exo regenerative effects on injured tendons [82]. Liu et al. found elevated p-SMAD2/3 and p-SMAD1/5/9 by Western blot in TSCs treated with ADSC-Exos. Moreover, after pretreated with SMAD2/3 inhibitor (SB431542,) or the SMAD1/5/9 inhibitor, the proliferation, migration, and tenogenic differentiation of TSCs were significantly suppressed, even after ADSC-Exo treatment. Tenogenic genes (TNMD, collagen I, and SCXA) were also significantly decreased in TSCs after pretreatment with SB431542 or dorsomorphin [57]. Similarly, Zhang et al. recorded significantly higher levels of p-AKT and p-ERK1/2 in tenocytes after a treatment of TSC-Exos mixed with gelatin methacryloyl. Pretreatment with the signal inhibitors before treatment with TSCs-Exos showed dramatically weakened effects of exosomes on tenocyte proliferation and migration [63]. The actual signaling pathways involved might be more complicated, and it is worthwhile to investigate these in future studies.

Moreover, most studies observed significantly higher expression of tenogenic genes at the injury site in the exosomal treatment group, including COL-1a1, Col3a1, SCX, and tenomodulin (TNMD), suggesting that exosomes derived from various sources promote tendon repair by enhancing tenogenesis on a gene level [59,61,67,74,83,84,85].

Exosomes not only enhance the proliferation and differentiation of TSCs/tenocytes directly, but also improve the stability of the tendon healing microenvironment. Thankam et al. found that the exosomes isolated from tenocytes and ADMSCs altered their levels of mRNA and related proteins in hypoxic environments (2% oxygen), with greater expression of matrix regenerative mediators (THSB1, NSEP1, ITIH4, and TN-C), offering a protective microenvironment for tendon healing [85]. Moreover, exosomes play a role in regulating the expression of COL-1a1, Col3a1, MMPs, and TIMPs, which is critical for creating a healthy extracellular matrix for tendon healing [63,67,73,74,83,86]. Hence, the exosomes promote tendon healing through balancing the remodeling of tendon ECM.

### 2.3. Bio-Engineered Exosomes on Tendon Repair and Regeneration

The biological properties of exosomes led to the idea of developing bio-engineered particles to either enhance or mimic their effects on tendon repair and regeneration. Based on the feature of exosomes on macrophage polarization, Chamberlain et al. applied exogenous human MSC-Exos on macrophages from healthy human peripheral blood, creating an M2-like phenotype (exosome-educated macrophages [EEMs]), and hypothesized that the bio-engineered exosome-educated macrophages can improve tendon repair [58]. The team used a mouse Achilles tendon healing model, and their data demonstrated significant improvement in tendon healing 14 days after tendon repair. The repair site showed increased biomechanical properties, numbers of endothelial cells, and the M2/M1 macrophage ratio in the EEM treatment group [58]. However, more investigation on dose-response and timing of injections needs to be fully explored in EEM-induced tendon treatment. Moreover, IFNγ-primed ASC-Exos demonstrated more effective suppression of NF-kB, indicating that inflammation-stimulated exosomes may have greater anti-inflammatory abilities [61]. Based on the finding that miR-29a-3p increased significantly via PTEN/mTOR/TGF-β1 pathway activation in HUMSC-Exo-treated tendons, Yao et al. applied an miR-29a-3p-specific agonist to engineer HUMSC-Exos. They found the gain effects on tendon healing were amplified after treatment of engineered exosomes that overexpress miR-29a-3p [82]. The same team developed another miR-21a-3p engineered HUMSC-Exos that inhibited tendon adhesion both in vitro and in vivo [68].

One major concern of exosomal treatment is that exosomes can only partially reach the targeted sites via passive diffusion. Common carriers used to deliver exosomes include hydrogel, fibrin glue, and gelatin methacryloyl [59,62,63,74,82,84,87,88]. Specifically, Liu et al. reported their experience in treating Achilles tendinopathy with nitric oxide nanomotor-driving exosome-loaded microneedles (EXO/MBA-loaded MN) to improve the efficiency of exosome delivery and permeation. The EXO/MBA-loaded MN patch group demonstrated notably more efficient tendon healing than the single exosome injection group [71]. Further studies on evaluating the physiological and mechanical properties of different delivery systems exosomes in the long-term are necessary.

A group from Mayo Clinic has recently developed a purified exosome product (PEP) from human plasma platelets as the first room-temperature-stable exosome product. The team added PEP both in vitro and in vivo in canine tendons, and achieved successfully enhanced tenocyte proliferation and tendon repair [89]. In a follow-up study, PEP was added on a TISSEEL patch (fibrin sealant) carrier and tested in a canine ex vivo model. Promisingly, this novel engineered exosomal patch can stably release particles over two weeks [74]. More organized and denser collagenous tissue and fewer inflammatory cells were also displayed at the tendon-bone interface after rotator cuff repair in the TISSEEL–PEP group [90]. Wellings et al. demonstrated significantly fewer external adhesions and improved mechanical functions with the application of PEP loaded onto a collagen scaffold, indicating that the off-the-shelf PEP product favored intrinsic healing instead of extrinsic healing [91].

Currently, the research on the effects of exosomes on tendon repair and regeneration is still scarce [6,66]. Studies involving injured tendinopathic tissues should be encouraged, as using tenocytes isolated from healthy tendons can be limited in their translation to injured tendon tissues.

## 3. Exosomes in Skeletal Muscle Repair and Regeneration

### 3.1. Skeletal Muscle Exosomal miRNAs

During myogenesis, the activation of muscle-specific transcription factors and the reprogramming of skeletal myogenesis genes are necessary for precursor cells to differentiate into myogenic lineages and form new myofibers [48,49]. In recent years, miRNAs have been determined to play a fundamental role in directing and modulating satellite cell/myoblast differentiation [5,49,66,92,93,94]. Mice with reduced muscle miRNAs (eliminated *Dicer* activity, specifically in muscles) showed decreased skeletal muscle mass and increased myoblast apoptosis, as well as abnormal myofiber morphology [95]. miRNAs circulating in the blood are sensitive to RNase and the oxidative extracellular environment [11,96]; thus, they are normally bound to proteins or protected by exosomes [97,98].

Unlike their parental cells, which are enriched in rRNA, exosomes are comparatively more abundant of miRNAs [11,99]. Myo-miRs are seen to be elevated in damaged muscle cells. However, many of these miRNAs later decline, and are only detectable in extracellular vesicles, indicating their active and selective packaging and sorting in response to muscle fiber damage [48,100,101]. More interestingly, vesicle-associated myo-miRs differ between dystrophy-associated damage and eccentric muscle damage, further suggesting a context-specific packaging of miRNA into the exosomes in response to tissue damage [102]. The way in which myo-miRs are selectively loaded to exosomes is poorly understood. However, investigations into exosomal miRNA cargo and sorting mechanisms can broaden the therapeutic options in muscle regeneration research.

### 3.2. Angiogenesis in Skeletal Muscle Regeneration

The biological actions of circulating angiogenic growth factors are enhanced due to the involvement of exosomes, which display a high capacity of delivering miRs, proteins, and other particles targeting cells with high angiogenic activity [103].

Nakamura et al. first found that MSC-derived exosomes enhanced angiogenesis in vitro and in vivo [97,104]. The team treated human umbilical-vein endothelial cells (HUVECs) with DMEM, MSC-conditioned medium, MSC-Exo suspension in DMEM, or exosome-depleted MSC-conditioned medium, and data showed a significantly increased number of migrated HUVECs accompanied by greater tube formation after MSC or MSC-Exo administration. The capillary density was found to be significantly higher in the MSC-Exo group following intramuscular injection of MSC-Exos in vivo [104]. Similarly, Cavallari et al. treated endothelial cells with serum-derived extracellular vesicles, and saw enhanced capillary-like (tube-like) structure formation in vitro [105]. In addition, they tested EVs in an acute hind limb ischemia mouse model, and found that serum-derived EVs can improve vascular remodeling and minimize muscle damage in vivo. Functional scores of the affected limb were significantly higher, and necrosis in the gastrocnemius muscle was almost completely prevented following EV treatment [105]. While Nakamura et al. demonstrated that miR-494 was the major protective mediator in exosomes that enhance HUVEC migration, Cavallari et al. identified TFG-b1 signaling cascade as a relevant pathway. In a mouse cardiotoxin-induced skeletal muscle damage model, Lo Sicco et al. implanted matrigel plugs containing MSC-EVHypo and MSC-EVNormo. Both groups induced the formation of tube-like endothelial structures surrounding the plugs, and upregulated the expression of angiogenic factors, including platelet and endothelial cell adhesion molecule (PECAM) and VEGFA. However, MSC-EVHypo, which are EVs from MSC exposed to hypoxic conditions, demonstrated higher expression of pro-angiogenic factors and more enhanced angiogenesis in the injured tibialis anterior muscles than MSC-EVs released under normoxia [106]. Figliolini et al. further analyzed the content of ASC-EVs for detecting genes involved in angiogenesis, and discovered enrichments in angiopoietin, VEGFA, HGF (hepatocyte growth factor), IGF1 (insulin-like growth factor-1), and EGF (epidermal growth factor) mRNA [107]. Mellows et al. observed similar angiogenic effects of EVs from human amniotic fluid stem cells (AFSCs), and reported an amplified number of capillaries in the CTX mouse model [108]. miR126 and miR23a, which are known to target angiogenesis-related pathways and restore vessel integrity, were also found to be abundant in MSC-EVs, and can be actively triggered by MSC-EVs [106,109]. More recently, Kato et al. confirmed the molecular mechanism behind the promising therapeutic effects of mesenchymal stem cells on angiogenesis in the injured muscle. They applied FW4869, which is a biogenic inhibitor of exosomes, to ADSCs, and revealed impaired ADSC-induced angiogenesis with reduced expression of miR-21, miR-27b, miR-322, and let-7i [110].

Interestingly, two studies reported suppressed or no angiogenic functions with EV administration. Wang et al. noticed angiogenesis was partially reduced by ASCs-Exos, which is contrary to most studies [111]. In addition, Mitchell et al. noted increased angiogenesis by secretomes of ADSC, but the EV fraction lacked angiogenic factors (VEGF, SPRED1, VECAM1, and IGF1). Moreover, the EV fraction held low levels of miR-494 [109]. Exosomes promoting angiogenesis in other types of tissues have shown to be concentration-dependent. The inconsistent effect of exosomes on angiogenesis in skeletal muscles is perhaps due to different sources of exosomes, different carriers, different concentrations/doses, etc. Furthermore, there may be multiple routes involved in the promotive features of angiogenesis in muscle regeneration, and potential studies may focus on differential secretome generation protocols.

### 3.3. Immunomodulation in Skeletal Muscle Regeneration

Similar to their influences on modulating inflammation in injured tendons, exosomes promote macrophage polarization and inhibit inflammation in skeletal muscle in a comparable manner. Cavallari and his group first described the impact of EVs on inflammatory cells, and reported that the infiltrate of inflammatory cells in ischemic gastrocnemius muscle was almost completely prevented by serum-EVs [105]. However, the team did not explore the mechanism.

Proinflammatory M1 macrophages are responsible for the removal of cellular debris, followed by anti-inflammatory/promyogenic M2 macrophages 3–7 days after the injury. The latter activates myogenic precursors, and leads to fusion and the formation of muscle fibers, which are especially important in muscle repair and regeneration. Lo Sicco et al. first described the effects of MSC-EVs on macrophage polarization. In vitro, the M1 to M2 phenotype switch was elicited in bone marrow-derived macrophages internalized with MSC-EVs. In vivo, a downregulation of IL-6 and Nos2 (a marker of innate and classical macrophage activation), as well as a significant upregulation of Arg1 and Ym1(markers of alternative macrophage activation), were recorded in cardiotoxin-injured skeletal muscle treated with MSC-EVs, indicating the ability of EVs to switch macrophages into the anti-inflammatory and healing phenotype [106]. More recent studies further demonstrated the ability of BMSC-Exos and myoblast-Exos to significantly reduce the expression of proinflammatory factors (iNOS, TNF-a, IL-1b, and IL-6), and increase the expression of anti-inflammatory factors (TGF-b andIL-10), converting M1 macrophages into M2 phenotype, both in vitro and in vivo [112,113,114]. Moreover, injured pubococcygeal muscles treated with M2-Exos received significantly accelerated muscle regeneration, whereas M1-Exos had little effect on myoblast differentiation [115].

Mitchel et al. confirmed that the anti-inflammatory effects of MSCs-secretomes primarily come from EV fraction, showing a significant decrease in the amount of NF-κB p65 in the nuclei of treated cells. The total secretome fraction only resulted in a non-significant reduction of nuclear p65 [109].

Interestingly, in muscular dystrophy, exosomal miRNAs reduce macrophage expression of TLRs, and enhance the take-up of these cells and their cargoes without immune stimulation [116,117]. Such findings raise the intriguing possibility that exosomes perhaps act in coordinated ways to fine-tune their cellular signaling in muscle repair and regeneration [102].

### 3.4. Satellite Cells Differentiation

During skeletal muscle repair, activated satellite cells differentiate into myoblasts. Then, these myoblasts undergo terminal differentiation, and eventually form multinucleated myotubes [118,119].

A hallmark of exosomes in muscle regeneration was reached by Nakamura et al. The group not only investigated the beneficial effect of EVs on angiogenesis, but also demonstrated their role in myogenesis with C2C12 myoblasts. The authors noted increased muscle cross-sectional areas and decreased fibrotic areas after MSC-EV injection [104]. Additionally, they evaluated the relation of miR-494 to myogenesis by transfecting C2C12 myoblasts with miR-494. The fusion indices of miR-494 transfected myoblasts were significantly higher than those transfected with siNega [104]. Forterre and his team reported comparable findings that muscle-related exosomes can promote myogenesis by delivering miRNAs between proliferating myoblasts and maturing myotubes [120,121,122]. Cho and his group isolated exosomes from HSkMs during differentiation into myotubes, and revealed their enrichment of various myogenic factors, including insulin-like growth factors, hepatocyte growth factor, fibroblast growth factor-2, and platelet-derived growth factor-AA, indicating that exosomes greatly contribute to myogenic differentiation. They later treated a muscle laceration mouse model with HSkM-Exos, and observed enhanced muscle regeneration with significant expression of myogenic proteins and genes [98]. Luo et al. also published that exosomes derived from C2C12 myoblasts boost myoblast proliferation/differentiation [113]. Byun et al. mentioned that the improved proliferation of muscle satellite cells after exosomal treatment was dose-dependent, and that 25 µg/mL was optimal. Comparably, injection of exosomes isolated from BMMSCs showed accelerated recovery of muscle contractile function [123]. Treatment activated the formation of new myofibers, and modulated the expression of myogenic genes [123]. The promising effects of exosomes on promoting satellite cell differentiation and upregulating myogenesis genes in injured skeletal muscle have been established thoroughly in several studies. However, the underlying pathways have hardly been reported. NRG-1 is a possible signal that drives angiogenesis and muscle protection in ASC-EVs [107].

The genes involved in muscle regeneration, including MYOG, MYOD, myogenin, Pax7, and eMyhc, were reported to be significantly increased in exosome-treated satellite cells or injured muscle [106,107,121,123,124,125]. This was followed by an improvement in muscle function in vivo.

Exosomes not only enhance myofiber development but are also able to inhibit apoptosis of myoblasts, myocytes, and endothelial cells [107,111,126]. Li revealed that BMSC-Exos can prevent the decrease of myotube diameter induced by dexamethasone via the miR-486-5p/FoxO1 Axis [127]. They are also shown to delay the degenerative changes of the supraspinatus muscle in a rat model of massive rotator cuff tear [111]. This can lead to novel therapeutic ideas for rotator cuff tears.

### 3.5. Bio-Engineered Exosomes as a Potential Treatment in Limb Ischemia

The development of the biomaterials based on exosomes’ effects on myogenesis is very limited, suggesting that it is a field that has limitless possibilities in the future. Some studies have used uncommon sources of exosomes that are widely and easily available, such as human urine and bovine milk [125,128,129,130]. Human urine-derived stem cell EVs (USC-EVs) induced the proliferation of endothelial cells and C2C12 myoblasts in a mouse hind limb ischemia model [130]. Other labs tried to generate exosomes from transduced/induced vascular progenitor cells (iVPCs), and tested their angiogenesis in ischemic muscles in pilot studies [131].

Diabetic patients are prone to limb ischemia. Zhang et al. transfected ADSCs with GLO-1, which was proved to ameliorate the proangiogenic ability of ADSCs in diabetic hindlimb ischemia [132]. Unfortunately, the GLO-1-overexpressing-ADSCs (GADSCs) have an extremely low survival rate in vivo [126]. G-ADSC-Exos acted as a stable nanocarrier, and succeeded in improving neovascularization in ischemia hindlimb muscle (gastrocnemius, gracilis, and quadriceps muscles) under hyperglycemic conditions [126].

Another hot topic of applying exosomal cargo for muscle healing is the treatment of Duchenne muscular dystrophy [26,133,134]. The transfer of SOD1 and SOD3, or myostatin propeptide through exosomes can replace the mutated SOD1, and accelerate muscle regeneration [26,133].

## 4. Exosomes in Peripheral Nerve Repair and Regeneration

### 4.1. Wallerian Degeneration and Axonal Regeneration after PNI

Peripheral nerve injuries (PNI) are not uncommon. More than one million people are affected yearly worldwide [135]. Approximately 2–3% of all patients admitted to a Level I trauma center suffer from PNI [136]. These injuries result in life-long disability and significant socioeconomic consequences [137]. The most serious injury is neurotmesis, or complete nerve transection, resulting in limited nerve regeneration and functional recovery [138].

Following PNI, multiple cellular and molecular processes work in a coordinated fashion to repair the damaged nerves. These processes include Wallerian degeneration, Schwann cell (SC) activation, neovascularization, inflammatory responses, and neurite outgrowth [139]. Soon after PNI, Wallerian degeneration sets in [140], followed by macrophages digesting degenerated axonal and myelin debris [141,142]. SCs play a crucial role in peripheral nerve regeneration, including (i) enhancing cell proliferation and de-differentiation; (ii) the development of bands of Bungner; (iii) the secretion of neurotrophic factors; (iv) recruiting macrophages; and (v) the removal of the debris [143].

There are many studies on the physiological, pathological, diagnostic, and therapeutic aspects of exosomes in axonal regeneration after nerve injury [144]. Exosomes in the nerve system are secreted by neurons, neural crest cells, and other supporting cells, such as astrocytes, oligodendrocytes, microglia, SCs (Schwann cells), and endothelial cells [145,146]. In CNS, exosomes have shown a promising lead for diagnosis of disease progression, treatment, and drug delivery in neurodegenerative diseases [147,148]. In the last decade, numerous studies have shown the promising role of exosomes and their cargo in peripheral nerve regeneration [14,149].

Impacts of the exosomes on following the process of peripheral nerve regeneration have been widely studied [14,150]. Exosome cargo mainly includes signaling proteins, miRNAs, and mRNAs involved in intercellular communication, which is a vital aspect of neuronal development [151,152]. miRNA-132 in exosomes is demonstrated to play an important role in neurovascular communication [153,154]. Exosomes act as paracrine control particles, which act on target cells to generate a response [155].

### 4.2. Schwann Cell Activation and Functional Optimization in PNI

One of the most distinguished features of peripheral nerves is their high capacity of regeneration, which contrasts with the CNS. SCs, as the major cell type in PNS, play a dominant role in the regeneration process [156,157,158]. It was not until recently that exosomes and their impact on SCs have come into focus. Studies have demonstrated that exosomes from various sources can enhance peripheral nerve development and regeneration by activating SCs, regulating their differentiation and proliferation, and inhibiting their apoptosis, as well as optimizing their functions during remyelination [159,160,161,162,163,164,165,166,167,168,169,170,171].

Exosomes derived from SCs regulate SC activities via different mechanisms. One study demonstrated that exosomes released from differentiated SCs upregulated miRNAs, such as miR211, miR363, miR22-3p, and miR29a-3p, and inhibited SC migration, while undifferentiated SCs did not [172]. This is consistent with the finding that differentiated SCs need to be reprogrammed into repair Schwann cells (rSCs) to promote nerve regeneration [173]. Mao et al. found upregulation of c-JUN, Notch1, GFAP, and SRY -box 2 (SOX2), which are characteristic genes of dedifferentiation and repair phenotypes of Schwann cells, after administration of GMSC-derived EV, suggesting that GMSC-EVs directly reprograms Schwann cells into a repair phenotype [174]. Comparably, Rao et al. published that GMSC-Exos promoted Schwann cell proliferation in vitro, and then observed significantly enhanced thickness of myelin sheath after direct transplantation of GMSC exosome-loaded chitin conduits to a rat sciatic nerve defect model in vivo [175]. Moreover, Wang et al. found that SC-Exos are involved in directing BMSCs differentiation towards Schwann cells. BMSCs showed significantly increased levels of Schwann cell-specific surface markers (S100, GFAP, Sox10, NGFR, and EGR2) compared with untreated BMSCs [169]. In type 2 diabetic mice, SC-Exos promoted the migration of SCs, which is normally challenged by the high glucose condition [176]. Fan et al. also observed increased myelin sheath thickness and improved sensory nerve conduction velocities in diabetic mice [177]. These findings are helpful for the further development of diabetic neuropathy treatments that can abolish high- glucose-inhibited cell migration and proliferation.

Some studies suggest that harvesting exosomes from SCs need to sacrifice normal neural tissues [150,167]. Fortunately, exosomes derived from other cell types also established beneficial impacts on peripheral nerve regeneration. ADSC-Exos are the most studied. Chen et al. noted that SCs internalized ADSC-Exos, and promoted their proliferation, migration, myelination, and axonal regrowth both in vitro and in vivo [165]. Yin et al. reported inhibited autophagy of SCs by ADSC-Exos via miR-26b-regulated downregulation of Kpna2, leading to regeneration of the myelin [159]. Bucan et al. and Liu et al. also observed enhanced SC proliferation induced by ADSC-Exos [178,179]. Moreover, Liu et al. noticed the anti-apoptotic effect on SCs from ADSC-Exos, since they upregulated the Bcl-2 expression and downregulated the Bax expression in SCs [179]. Haertinger et al. established that ADSC-EVs effects on SCs are time and dose dependent. Interestingly, they found, through live cell imaging, that ADSC-EVs were preferentially internalized at SC processes where the EVs were transported towards the cell nucleus, providing future approaches for therapeutic exosome intake [170]. hUCMSC-EVs also show effects of enhancing SCs proliferation in the sciatic nerve [171,180].

### 4.3. Neurite Outgrowth in PNI

Axons—specialized projections of neurons—are another major component of PNS. There is accumulating evidence that exosomes promote neurite outgrowth following PNI [171,176,178,181,182,183,184,185,186,187,188,189,190,191,192].

After injury, the proximal regenerating axon develops growth cones that communicate with the surrounding microenvironment. These growth cones promote SC migration [193,194] and SC de-differentiation, which depends on axonal contact [143,195,196,197]. These dedifferentiated SCs, in return, modulate various cellular and molecular processes, and enhance axon regrowth via exosomes [198]. A landmark study by Lopez-Verrilli et al. showed that SC-Exos were taken up by axons, and subsequently augmented neurite outgrowth in vitro [199,200]. Of note, SC-Exos increased the growth rate of dorsal root ganglion cell axons from 0.44-mm/day in controls to 0.61-mm/day [200]. The team confirmed these findings in vivo in a crush injury model, and recorded enhanced axonal regrowth (neurites which were two times longer) and improved nerve function using a pinch test. Furthermore, they determined that exosomes suppressed GTPase RhoA activity, which impeded the neurite growth in the SC-Exo-treated group. Lopez-Leal et al. further explained that the increase of neurite growth after SC-Exos treatment was driven by miR-21 (exosome cargo), which downregulated PTEN and PI3-kinase activation in neurons [173].

In addition, Bucan et al. demonstrated that adipose MSC-Exos enhanced neurite outgrowth, both in vivo and in vitro, via multiple neurotrophic factors that support neural survival and axonal growth, inducing glial cell-derived neurotrophic factor (GDNF), fibroblast growth factor-1 (FGF-1), brain-derived neurotrophic factor (BDNF), insulin-like growth factor-1 (IGF-1), and nerve growth factor (NGF) transcripts [178]. The levels of the expression of endogenous neurotrophic factors reflect the regenerative capacities of axotomized neurons and denervated Schwann cells. The higher the levels, the more powerful the support for neuron regeneration. Consistently, Rau et al. observed significantly increased expression of NGF and GDNF in the nerve segments of crush injury mice after ADSC-Exos treatment, compared with control mice [168].

Interestingly, there is also evidence that Schwann cells formed a vesicle-like structure providing labeled ribosomes that were budding from the Schwann cell to the axon after an injury [198]. Ribosomal proteins have been detected in SC-Exos [201]. Axons rely on the local translation of proteins from mRNAs that are essential for nerve regeneration, such as cytoskeletal proteins, but the axonal transport of proteins is relatively low. Hence, supporting axons with ribosomes from via exosomes could support the local protein synthesis, and achieve immediate regeneration response after an injury [198].

### 4.4. Immunomodulation in PNI

As with any tissue injury, PNI is associated with an inflammatory reaction facilitating both Wallerian degeneration and nerve regeneration. Neuroinflammation plays a crucial role in recovery from PNI. MSC-derived exosomes regulate inflammatory reactions in wound healing, bone repair, and cardiac tissue [202,203,204,205]. Studies have shown that axonal regeneration after PNI is not only mediated by SCs, but also largely by macrophages [167,206].

In the study of Ma et al., it was noted that EVs can migrate to nerve defects and induce immunosuppression by decreasing IL-6, IL-1β, and IL-10 [183]. Other exosome studies have shown the immunomodulatory role of exosomes by regulating IL-1, αB-crystallin, and galectin-1 [175,207,208].

The effect of exosomes on macrophage polarization in PNI is similar to their effect in tendon and skeletal muscle repair. In diabetic mice, Fan et al. reported that MSC-Exos diminished inflammatory response by enhancing M2 phenotype, and thus helped to treat peripheral neuropathy [177]. Simeoli et al. established a dysregulation mechanism of nerve repair, in which sensory neuron-derived exosomes phagocytized by macrophages expressed increased miR-21-5p, which promoted M1 phenotype, leading to disrupted nerve regeneration. They suggested that miR-21-5p can be considered as a target cargo for improving nerve repair [206].

### 4.5. Bio-Engineered Exosomes as a Potential Treatment in PNI

The achievements that have been made in this field are exciting. Yang et al. utilized ADSC-Exos as cargo for delivering neurotrophic factors (NTFs), such as neurotrophin-3 (NT-3). They encapsulated NT-3 mRNA ADSC-Exos. Then, they applied these engineered exosomes to a rat sciatic nerve defect model by loading them into nerve guidance conduits (ExoNT-3-NGC). After NGC implantation, nerve regeneration and the functional recovery of gastrocnemius muscles were significantly improved, compared with the control group (Exo-empty-NGC) [209]. Fan et al. engineered MSC-Exos with miR-146a, and obtained amplified therapeutic effects on DPN in diabetic mice.

Moreover, Yu et al. generated an artificial nerve graft incorporated with extracellular vesicles derived from skin-derived precursor Schwann cells (SKP-SC-EVs), and bridged a 10-mm long sciatic nerve defect in rats. Compared with silicone conduits and autografts, the newly developed nerve grafts significantly accelerated the recovery of motor, sensory, and electrophysiological functions by facilitating outgrowth and myelination of regenerated axons, as well as alleviating denervation-induced atrophy of target muscles [210].

Purified exosome product has promising effects on improving outcomes of peripheral nerve reconstruction. Before applying the product, Ikumi et al. reconstructed the sciatic nerve defect with the reversed nerve autograft in a rat model. The PEP group demonstrated significantly larger axon diameter and elevated GAP43 and S100b levels compared with other groups after the surgery, indicating its beneficial effects on nerve autograft [184].

## 5. Future Directions

Presently, exosomes have demonstrated promising therapeutic effects on soft tissue (tendon, skeletal muscle, and peripheral nerve) repair and regeneration. These nanoparticles and their mimics can exert similar therapeutic effects as those achieved by cell therapies, but avoid many disadvantages. It is encouraging that most of the experimental evidence suggests the effectiveness and safety of using exosomes. However, studies were only performed in pre-clinical models. As we can see from this review, the development of exosomal treatments is still at a very early stage of research, and little clinical application has been accomplished. One revolutionary advance is the newly developed PEP from Mayo Clinic (Table 1), which has already undergone clinical trials (human; in phase I clinical trial; ClinicalTrials.gov: NCT04664738, https://clinicaltrials.gov/ct2/show/NCT04664738, accessed date: 16 March 2021) in tendon repair [74,89,90], peripheral nerve regeneration [184], vaginal tissue regeneration [211], and myocardial infarction recovery (human; in phase I clinical trial; ClinicalTrials.gov: NCT04327635, https://clinicaltrials.gov/ct2/show/NCT04327635?cond=Myocardial+Infarction&cntry=US&state=US%3AMN&city=rochester&draw=7&rank=12, accessed date: 2 November 2021).

In addition, related signaling pathways of exosome functions are still not well established. Results from different studies are inconsistent. Exploring the mechanisms of the promotive effects of exosomes on soft tissue regeneration is another time-worthy aspect. Moreover, there are currently no standard techniques for exosome isolation and purification, nor a standard approach for exosome administration, nor exosome carriers. Establishing the effective route for exosomal injection as well as standard techniques for isolation, qualification, and purification is essential for the clinical translation of exosome use. Finally, although there are many breakthroughs in understanding the mechanism of action of exosomes and their advantages over cell therapies, few studies have evaluated the safety and side effects of exosomal treatments. For instance, plasma-derived exosomes may deliver molecules of diseased tissues and drugs with high toxic potential [212]. Tumor-derived exosomes can deliver chemotherapeutics to the recipient cells both in vitro and in vivo [213,214]. Cossetti et al. also demonstrated that exosomes are capable of transferring substances of tumor cells to recipient cells [215]. Further investigations are needed to determine safer sources of exosomes. To address concerns regarding the safety of exosomes, a stepwise approach would be appropriate [212]. The first step is to look for a safe and effective source of these therapeutic exosomes. The next step is to determine the mechanisms through which exosomes exert their therapeutic effects. The final step could be the modification of the exosomes to make them safe, such as by obtaining them from the same patient and loading these exosomes with already known cargo, and re-infusing the patient with these modified exosomes. They can also be modified indirectly by isolating and manipulating, for instance, immature dendritic cells (iDCs) or mesenchymal stem cells (MSCs), with the aim of producing exosomes bearing therapeutic molecules. These engineered exosomes may be administered to the patient [212].

**Figure 1:**
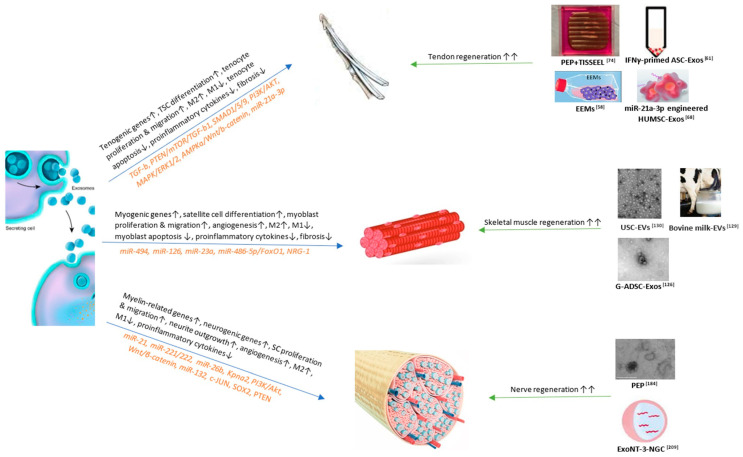
TSC, tendon stem cell; PTEN, phosphatase and tensin homologue; mTOR, mechanistic target of rapamycin; TGF, growth factor beta; PI3K, phosphatidylinositol 3-kinase; AKT, protein kinase B; MAPK, microtubule associated protein kinase; ERK, extracellular signal-regulated kinase; AMPK, adenosine monophosphate-activated protein kinase; Wnt, wingless-related integration site; miR, microRNA; PEP, purified exsomal product; IFN, interferon; ASC, adipose stem cell; EEM, exosome-educated macrophage; HUMSC, human umbilical cord mesenchymal stem cell; FoxO1, forkhead box O1; NRG-1, neuregulin 1; USC, urine-derived stem cell; G-ADSC, adipose-derived stem cells overexpressing glyoxalase-1; SC, Schwann cell; Kpna2, Karyopherin Subunit Alpha 2; SOX2, sex determining region Y-box 2; NT-3, neurotrophin-3; NGC, nerve guidance conduit. References: [58,61,68,74,126,129,130,184,209].

All in all, exosomes are very promising therapies in the regenerative medicine, and accelerating their application to clinical trials is the next step. Figure 1. The role of exosomes in soft tissue repair and regeneration (tendons, skeletal muscles, and peripheral nerves).

Exosomes promote soft tissue repair and regeneration through different mechanisms and signaling pathways. The currently published important mechanisms and pathways and related miRs are displayed in Figure 1 (left). Based on the mechanisms, many studies have established bio-engineered exosomal products that show benefits to soft tissue wound healing as listed in Figure 1 (right). Among them, PEP can significantly enhance both tendon [74,91] and peripheral nerve [184] regeneration, but has not been studied on skeletal muscles. Future studies regarding PEP’s effects on muscle repair can be useful. Bovine milk [129] and urine-derived exosomes [130] provide the most readily available resources; however, they have only been studied on skeletal muscles. Further studies regarding the effects of bovine milk and urine-derived exosomes on the tendon and peripheral nerve tissue repair can be valuable [128].

## Figures and Tables

**Table 1:** The application of the purified exosome product in soft tissue repair and regeneration.

Type of Tissue	Carrier for Delivery	Outcomes	Ref.
Flexor tendon	PEP solution in vitro	Enhanced tenocyte proliferation ability, high level of tendon-related genes expression, increased total collagen deposition.	[89]
Flexor tendon	TISSEEL (patch)	The patch can stably release effective exosomes over two weeks; higher failure load strength, smaller healing gap, increased expression of tendon-related genes, reduced inflammatory response, increased formation of type III collagen.	[74]
Achilles tendon	A type 1 collagen scaffold	Improved mechanical functions, lower adhesion grade.	[91]
Rotator cuff	TISSEEL	Promoted migration and proliferation of osteoblasts and tenocytes in the repaired supraspinatus tendon, accelerated healing of the rotator cuff.	[90]
Sciatic nerve	Fibrin glue	Better isometric tetanic force, larger average axon diameter of the peroneal nerve, upregulated GAP43 and S100b gene expression.	[184]

PEP, purified exosomal product; GAP43, growth associated protein 43; S100b, S100 calcium-binding protein B.

## Data Availability

The data presented in this study are available on request from the corresponding author.
